# Merkel cell carcinoma

**DOI:** 10.1186/1477-7819-4-7

**Published:** 2006-02-08

**Authors:** Virve Koljonen

**Affiliations:** 1 Department of Plastic Surgery, Helsinki University Hospital, Helsinki Finland

## Abstract

**Background:**

Merkel cell carcinoma (MCC) is an unusual primary neuroendocrine carcinoma of the skin. MCC is a fatal disease, and patients have a poor chance of survival. Moreover, MCC lacks distinguishing clinical features, and thus by the time the diagnosis is made, the tumour usually have metastasized. MCC mainly affects sun-exposed areas of elderly persons. Half of the tumours are located in the head and neck region.

**Methods:**

MCC was first described in 1972. Since then, most of the cases reported, have been in small series of patients. Most of the reports concern single cases or epidemiological studies. The present study reviews the world literature on MCC. The purpose of this article is to shed light on this unknown neuroendocrine carcinoma and provide the latest information on prognostic markers and treatment options.

**Results:**

The epidemiological studies have revealed that large tumour size, male sex, truncal site, nodal/distant disease at presentation, and duration of disease before presentation, are poor prognostic factors. The recommended initial treatment is extensive local excision. Adjuvant radiation therapy has recently been shown to improve survival. Thus far, no chemotherapy protocol have achieved the same objective.

**Conclusion:**

Although rare, the fatality of this malignancy makes is important to understand the etiology and pathophysiology. During the last few years, the research on MCC has produced prognostic markers, which can be translated into clinical patient care.

## Background

### The Merkel cell

The Merkel cell (MC) was first described by the German histopathologist Friedrich Sigmund Merkel in his classic article published in 1875: Merkel F: "Tastzellen und Tastkörperchen bei den Haustieren und beim Menschen" [1]. He demonstrated the existence of touch cells in the snout skin of pigs, calling them "Tastzellen" because of their putative function in the touch sensation. Merkel postulated that the cells acted as mechanoreceptors in all animals.

### Distribution of Merkel cells

**Figure 1:**
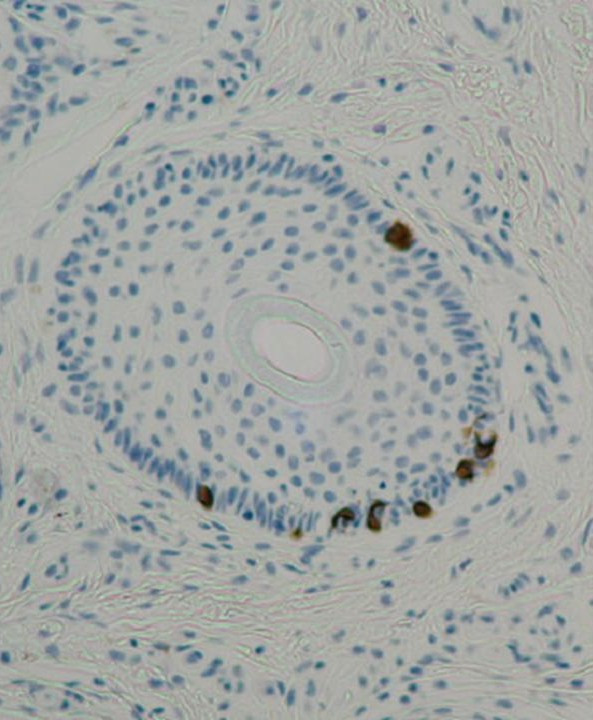
Solitary Merkel cells in the normal skin, expressing cytokeratin-20.

MCs are normal constituents of the basal layer of the epidermis and the follicular epithelium [2] (Figure 1). They are scarce in normal skin, but are present in high numbers and form clusters in areas of sensory perception. In close association with primary nerve endings in the skin, they form the MC-axon complex.

On light microscopy, MCs are large, oval, amphophilic, clear cells situated in the basal or suprabasal layer of the epidermis. They are not easily distinguished from other nonkeratinocytic epidermal cells, e.g. melanocytes and Langerhans cells, by light microscopic immunohistochemistry. Special techniques such as immunohistochemistry, electron microscopy or transmission electron microscopy are therefore required for their identification.

### Function in developing embryo and adult

In human development, MCs can be detected in the epidermis in the 8^th ^gestational week [3]. In the developing embryo, MCs are thought to be involved in the formation of the subepidermal nerve plexus [4,5], and in the formation and proliferation of eccrine sweat glands and hair follicles [6]. In the adult MCs are thought to act as slow acting type-I mechanoreceptors. Together with sensory nerve endings, they form MC-axon complexes that are activated by steady skin indentation [7]. The function of MCs in this complex is, however, enigmatic. Two possible functions have been proposed: either they may act as attracters of developing or regenerating type I nerve fibres [8] or they may have neuromodulatory or neuroregulatory functions in the basal epidermis, such as keratinocyte proliferation stimulation, maintenance of the differentiation of keratinocytes, or the release of bioactive substances to subepidermal structures [9].

### Origin of the Merkel cell

The origin of the MC is still controversial. The cell may derive either from the epithelial cells of the epidermis or from the neural crest migrating to the epidermis during embryogenesis. In the 1980s and 1990s, the epidermal origin from keratinocytes with an aberrant differentiation [10] was the prevailing hypothesis [3,11-13], as suggested by the epidermal location of the MC, the expression of cytokeratins and the results of skin transplantation experiments [14,15]. Recent studies, however, have provided strong evidence in support of the neural crest origin [16,17].

#### Merkel cell carcinoma

The Merkel cell carcinoma (MCC) was first described in 1972, when Toker presented the first five cases under the name "trabecular carcinoma of the skin", assuming them to represent an eccrine, sweat gland-derived carcinoma [18]. In electron microscopic (EM) studies, Tang and Toker later identified dense-core neuroendocrine granules within the tumour cells, thus demonstrating their origin from MCs [19].

The "cell of origin" of MCC is, however, still speculative. There are morphological and biological similarities between the MC and MCC. The common presence of neuroendocrine granules [20] and the positive immunostaining for cytokeratin-20 (CK-20), for instance, provide evidence for MC origin [21], although this may indicate differentiation rather than origin. However, certain differences also exist between the MC and MCC, such as the fibrous whorls and neurofilaments that are seen in MCC but not in normal MCs. Another argument against the MC as the cell of origin for MCC is that mitoses have not been detected in human MCs [3,22]. There is a contradiction between the location of the cell in the skin in MCs and in MCC, the tumour practically always involves the dermis, sparing the epidermal structures.

Toker called the first five cases of MCC "trabecular carcinoma of the skin." This name derived from the trabecular architecture of the tumour, which is the most characteristic, but the uncommon, configuration of three histological patterns. Over the years, the name of this tumour has been the subject of lively discussion. Several names and synonyms have been proposed; either based on histological features or derived from the term "Merkel cell". These include derivatives such as "Malignant Merkel-cell tumour" [23], "Merkel cell tumour" [24] or "Merkel cell tumour of the skin" [25]. It was not until the mid 1980s that the term "Merkel cell carcinoma" was established. In the year 1980, Johannessen and co-workers seem to have used this term for the first time [26]. The term "Primary neuroendocrine carcinoma of the skin" was coined to reflect the pathophysiology of the disease.

### Histology

The histology of MCC is typical of small round blue cell tumours, an entity that includes a wide variety of highly malignant tumours: the Ewing family of tumours, olfactory neuroblastoma (esthesioblastoma), rhabdomyosarcoma, neuroblastoma, lymphoma, desmoplastic small cell tumour, osteosarcoma, small cell lung carcinoma (SCLC), small cell melanoma and mesenchymal chondrosarcoma [27,28].

Histologically, MCC can be classified into three distinct subtypes [29-31]. In haematoxylin-eosin staining, typical histology of the MCC is small blue cells with sparse cytoplasm. Nuclei are medium-sized. Mitoses are abundantly and the tumour expands frequently to subcutaneous tissue. Chromatin is displayed in typical salt and pepper pattern.

The classification to the subtypes is usually reserved for the anatomical studies. The first of these is the **trabecular subtype**, described originally by Toker[18]. This is the least frequent histological pattern. Cells are arranged in distinctly organoid clusters and trabeculae with occasional ribbons. This type of tumour usually occurs adjacent to adnexal structures, particularly hair follicles.

The **intermediate subtype **is the most frequent histological subtype [32]. It exhibits a solid and diffuse growth pattern. Cells are less compactly arranged, and the cytoplasm is less abundant than in the trabecular type. Mitoses and focal areas of necrosis are frequent. These tumours usually arise adjacent to adnexa, but may invade the epidermis. The clinical behaviour is more aggressive than that of tumours of the trabecular type. The **small cell type **mimics small cell tumours of other sites, e.g. SCLC [33]. The tumours arise in the dermis and appear as solid sheets and clusters of cells. The clinical behaviour of this subtype appears to be as similar as the small cell tumours of other origins.

### Diagnosis

**Figure 2:**
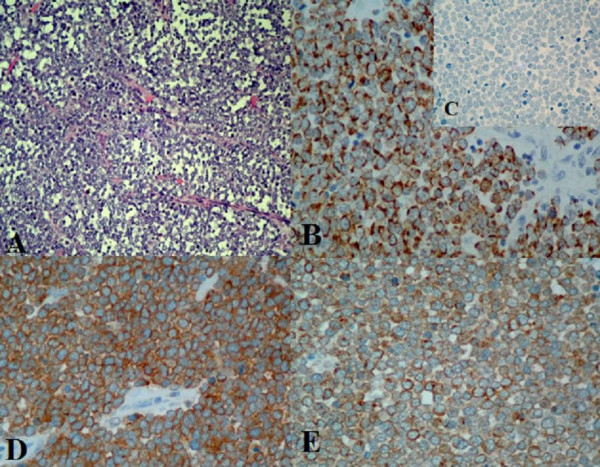
Immunohistochemical staining of primary Merkel cell carcinoma for differential diagnosis and neuroendocrine differentiation. A. Hematoxylin-eosin staining, the tumour cells have round nuclei, original magnification 200×. B. Positive cytokeratin-20 staining, showing typical punctate pattern of immunostaining, original magnification 400×. C. Negative staining for Thyroid-transcriptor factor-1, original magnification 400×. D. chromogranin -A staining, original magnification 400× and E. synaptophysin staining, original magnification 400×.

Diagnosis is based on typical histology representation on haematoxylin-eosin -stained slides together with the results of immunohistochemistry [32]. A typical histological finding in MCC is the presence of tumour tissue within the dermis with repeated extensions to underlying subcutaneous tissue (Figure 2). The epidermis, papillary dermis and adnexal structures are not usually involved. Cytological features include sparse cytoplasm with uniform, monotonous medium-sized nuclei and abundant mitoses [34]. All these cytomorphological features can be present as well in fine needle aspirates [35]. EM studies show that the ultrastructure of neuroendocrine granules of the tumour is similar to that of normal MCs. The most consistent findings are the aggregation of intermediate filaments in a paranuclear location and the existence of membrane-bound dense core granules. Only 10 % of all MCC tumours are intraepidermal [36].

### Immunohistochemistry – differentiation

The tumour expresses both epithelial and neuroendocrine markers, and thus exhibits both epithelial and neuroendocrine differentiation.

#### Epithelial differentiation

Of the intermediate filaments, MCC expresses low-molecular-weight cytokeratins (keratins 8,18,19 and 20), the simple epithelial type being the most marked [20,37]. The most important keratin in differential diagnostics is cytokeratin-20 (CK-20). CK-20 is a low-molecular-weight cytokeratin, in normal tissues it is only expressed in the gastrointestinal epithelium, urothelium, and MCs [38]. It has been shown that CK-20-positivity in MCC serves to distinguish it from pulmonary small cell carcinoma. Considered a sensitive and specific marker for MCC, CK-20 is helpful in efforts to distinguish between MCC and other malignant neoplasms, since it is not usually expressed in neuroendocrine carcinomas of other sites, such as SCLC [39,40] (Figure 2). Although up to 30 % of metastatic neuroendocrine carcinoma from lung might stain for CK-20, but they always stain for TTF-1, which is negative in MCC [41]. In some 5 – 25% of MCC cases, CK-20 is negative [42,43].

Tissue-specific transcription factors control cell determination and differentiation. Thyroid transcription factor 1 (TTF-1) is a tissue specific transcription factor expressed in epithelial cells of the thyroid and lung, as well as in certain areas of the brain [44]. TTF-1 is employed to differentiate between MCC and small-cell tumours [45,46], MCC is negative for TTF-1 [47] (Figure 2).

Combining TTF-1 with CK-20 provides a sound basis for diagnosis. Most adenocarcinomas from other sites (breast, lung, endometrium) and neuroendocrine carcinomas such as SCLC are essentially negative for CK-20 [40] while most are negative for CK-20, those from the lung are postitive for TTF-1. Table 1 presents the immunohistochemistry for the differentiation diagnosis of MCC.

**Table 1:** Immunohistochemical markers in the differential diagnosis of MCC.

	**CK-20**	**TTF-1**	**NSE**	**S-100**	**GrA**	**SYP**	**NFP**	**CD56**	**MAP-2**	**LCA**
**MCC**	+	-	+	-	+/-	+/-	+	+	+	-
**SCLC**	-	+	+/-	-	-/+	+	-/+	+	+	-
**MM**	-	-	-	+	-		-		+	-
**LGNEC**	+		+		+	+	-/+			
**Malignant lymphoma**	-	-	-	-	-		-/+			+

+ positive, +/- mostly positive, -/+ mostly negative, - negative. CK-20 cytokeratin 20, TTF-1 thyroid transcription factor 1, NSE neuron-spesific enolase, GrA gromogranin A, NFP neurofilament proteins, CD56 neural adhesion molecule, MAP-2 microtubule associated protein 2, LCA leukocyte common antigen SCLC small cell lung carcinoma, MM malignant melanoma, LGNEC low grade neuroendocrine carcinomas

#### Neuroendocrine differentiation

Due to neuroendocrine differentiation, MCC always stains positively for neuron-specific enolase (NSE), which is a general marker of neuroendocrine tumours [48-50]. CD56, or neural cell adhesion molecule (NCAM), has recently been demonstrated to be a neuroendocrine marker of the pulmonary neuroendocrine cell system as well as MCC [51,52]. Although, McNiff and co-workers recently reminded of the pitfalls in using CD56 marker for MCC diagnosis [53] Chromogranin A (CrA), a member of the chromogranin family, is a major protein that accounts for a large amount of the soluble matrix of neurosecretory granules. First isolated from chromaffin cells of the adrenal medulla, CrA is the most widely distributed marker of endocrine tumours [54]. MCC shows a focal, positive immunoreaction to CrA [55,56] (Figure 2). Synaptophysin is a transmembrane channel protein of small presynaptic vesicles. It is expressed in neuroendocrine and neural cells and diffusely in neuroendocrine system cells [57,58]. Both primary and metastatic neuroendocrine carcinomas are habitually synaptophysin positive [59,60]. MCC consistently shows positive immunoreactions to synaptophysin [56,61] (Figure 2).

Newer markers of neuroendocrine differentiation introduced for MCC are the microtubule-associated proteins (MAPs). These are the major component of the cytoskeleton family of proteins associated with the microtubule assembly of the central and peripheral nervous system [62,63]. Specific MAPs have been identified in specific cell types. MAP-2, for instance, is a highly sensitive and specific marker of neuroendocrine differentiation [62-64]. Liu *et al*, demonstrated MAP-2 expression in all MCC samples, even though CK-20 staining was negative [65]. In general, MCC shows a high degree of neuroendocrine differentiation. A higher expression of CrA and synaptophysin associated with more indolent behaviour [56].

### DNA copy number changes

Different types of chromosomal irregularities have been documented in MCC tumours and cell lines [66-68]. The most common aberrations engage chromosomes 1, 6, 11 and 13. Trisomy of chromosome 6 is a widely considered as recurrent aberration [69,70]. Trisomy of chromosome 1 appears to be typical of MCC [71]. In the chromosome 11 a partial trisomy has been documented [72] as well as complete trisomy [73]. In chromosome 13 loss was recognizable [72] in addition to LOH [74]. So far it seems that high-copy number amplifications for chromosomal subregions are a rare event in MCC [75]. Although DNA copy number changes have not been established as prognostic factor for MCC, primary tumours expressing DNA alterations were predominantly distinguished in large sized tumours, and risk of metastatic dissemination was three-fold compared to tumours with no DNA alterations [76].

#### Clinical presentation and staging

The clinical presentation of this tumour is rather non-specific. MCC is usually, at least at early stages of the disease, defined as a small painless erythematous intradermal mass, usually with no ulceration [77,78]. Especially small tumours in particular can appear somewhat benign, whereas large tumours have an unquestionably malignant appearance. Because of the rare nature of this neoplasm, it is often mistaken for more common skin tumours of epithelial origin. Most common presumptive clinical diagnosis is cyst.

There is no classification scheme for MCC such as there is for most carcinomas or sarcomas. At present, staging of most malignant tumours is based on the T(umour) N(odes) M(etastasis) classification (TNM classification of malignant tumours – 6th edition, 2002). Concerning the skin malignancies the TNM classification applies to carcinomas of the skin in general and malignant melanoma. In most published series MCC is not classified according to the TNM system.

Yiengpruksawan has, however, proposed a staging system that has been widely recognised in the treatment of MCC [77], which is presented in Table 2.

**Table 2:** Yiengpruksawan proposed staging system with treatment recommendation, SLNB = sentinel lymph node biopsy

Stage		Treatment Recommendation
I	Localized disease	Surgery: local excision > 2 m margins, SLNB Radiation therapy: adjuvant after resection
IA	≤ 2 m primary tumour	
IB	> 2 cm primary tumour	
II	Regional lymph node involvement	Surgery: local excision > 2 m margins, lymph node dissection Radiation therapy: adjuvant, primary site and lymph node region
III	Distant metastases	Radiation therapy: palliative use

#### Incidence and clinical behaviour of MCC

The reported annual incidence of MCC ranges from 0.2 to 0.45 per 100 000 [79,80]. Incidence has tripled since 1986:1986 0.15 per 100,000 and 2001 0.44 per 100,000 [81]. It is 100 times as rare as melanoma [82]. MCC is principally a disease of the Caucasian race. The annual age-adjusted incidence of MCC is 0.23 per 100,000 for whites and 0.01 for blacks [83]. The literature contains only single case reports of black Africans or African-Americans with MCC [84-86]. Both sexes are affected, though earlier studies showed a slight male predominance [87-89]. The number of reported patient series is, however growing, and recent investigations have shown an equal distribution of the sexes or even a minor female prevalence [90-92]. MCC mainly affects the elderly, the mean age at presentation being about 75 years [93,94]. Age-specific incidence was highest in the elderly, 4.28 per 100,000 in the 85+ age group [81] Only a few cases occur before the age of 50, and is usually related to immunosuppression [95].

MCC usually occurs in sun-damaged skin. The tumours are often found in close proximity to other lesions of actinically damaged skin, for instance, in cases of Bowen disease, squamous cell carcinoma, basal cell carcinoma, solar keratosis and lentigo maligna. The most common site is the head and neck region [96]. Pathogenetic factors such as UV irradiation may contribute to tumour development [68,97]. Miller and Rabkin have calculated that the incidence of MCC rises along with an increase in potential exposure to solar UVB. They found statistical significance between the incidence of MCC and UVB exposure [83].

The natural history of MCC is variable and dependent on the stage of disease at presentation [98]. Typically MCC has a tendency to progress rapidly, and in just a few months, the tumour may attain a large diameter. Therefore, early diagnosis and surgery are strongly advocated. Several studies established a close correlation between overall survival and tumour size. The overall survival rate is largely influenced by tumour size (≥ 2 cm) [99,100]. In some studies dense lymphocytic infiltrates have been associated with poor prognosis [101].

The typical clinical course of the disease is rapid progression of the primary tumour with early and frequent metastasis to the regional lymph nodes. The sentinel lymph node biopsy (SLNB) technique has provided information on the metastatic dissemination at the presentation. In a meta-analysis of 60 MCC patients, Mehrany and co-workers detected 33% of the patients having metastatic dissemination in the sentinel lymph node at the presentation [102]. Then again, Hohaus and co-workers in their 17 patients retrospective analysis reported only 3 (18%) of the patients at stage II at the presentation [103]. Allen and co-workers detected 5/26 (19%) of the patients having positive lymph nodes at the presentation [104].

Recurrences are frequent. Allen *et al*, at the Memorial Sloan-Kettering Cancer Center's identified 251 patients who had been treated between 1970 and 2002. The median time to recurrence was 9 months, and 102 patients (43%) recurred [98]. Primary site on the lower limb had an adverse effect on locoregional recurrencies [105].

Although the natural course of MCC is usually defined with metastatic spread, there are some reports in the literature (10 cases) of spontaneous regression [106,107]. Mori and co-workers have studied the apoptosis in eight cases of MCC [108] and found high apoptotic rate in MCC samples. Inoue et al have examined the mechanisms behind the spontaneous regression in seven MCC samples [109]. The TUNEL index and number of lymphocytes around the tumour nests was increased in the samples with spontaneous regression, furthermore the majority of the infiltrating lymphocytes were T-cells [109]. They concluded that apoptosis and local T-cell mediated immune response might be involved in spontaneous regression of MCC.

### Co-existing malignancies

MCC has been associated with other skin tumours (squamous cell carcinoma, basal cell carcinoma) and haematological (B-cell) malignancies [110-112]. In addition, adenocarcinomas of the breast and ovaries have been shown to have an association with MCC. Co-malignancies, whether they appear before, after or simultaneously with MCC, are associated with higher MCC-specific mortality [113,114].

### Immunosuppression: therapeutic and acquired

The incidence of MCC is abnormally high (8%) among immunosuppressed patients. Such patients are moreover younger, 49% being under the age of 50 [115,116]. Weakened immunity increases the risk of MCC; HIV patients, for example, have a 13.4 times increased risk of acquiring MCC [117].

#### Prognosis

The prognosis is rather poor. The 2-year survival rate is 30 – 50% [118,119], Kokoska included 35 and Linjawi 10 patients in his study. Agelli and Clegg have studied the epidemiology of MCC in the United States in a patient population of 1034 by using Surveillance, Epidemiology, and End Results Program. The 5-year survival rate in their study was 75%, 59% and 25% for localised, regional and distant MCC, respectively [120]. Overall survival (OS) is associated with the stage of disease at presentation. Female sex, localized disease, and younger age were positive predictors of survival [78,120,121]. The risk of recurrence or metastasis was 19 times as great in sentinel node biopsy-positive patients as in biopsy-negative patients (p = 0.005) [102].

Local recurrences are frequent, occurring in up to 44% of patients [30,88,93]. There have been some reports of lymph node metastasis alone, with no detectable primary tumour [122,123]. Depending on the length of the follow-up period up to 36% of the patients develop regional lymph node metastasis [124]. Distant dissemination is not infrequent, up to 40–50% of patients developing visceral metastasis [125-127], particularly to the lungs, liver and bone [128,129].

#### Surgical treatment

Because of the rarity of the tumour, there is multitude of treatment protocols. However, the surgical treatment seems to be the corner stone of the different treatment protocols.

Early, radical surgery is the recommended procedure for the treatment of primary MCC [92,130]. Margins of 2 – 5 cm are recommended for better local control. Nevertheless, consensus on the width of margins has not yet been reached. Yiengpruksawan and co-workers, O'Connor and Brodland reported better local control with margins of > 3 cm [77,131]. Then again, there have been reports that larger free margins confer no advantage on survival [124]. Gillenwater and co-workers had sixty-six head and neck MCC cases included in their study, but only eighteen patient's data was sufficient for the statistical analysis. They state themselves that the small patient population in their study might explain this result. Because lymph node metastases develop in approximately 50% of patients in the course of the disease, some authors recommend prophylactic lymphadenectomy in all patients [97,118]. Then again, the patient materials are small: Lawenda had nine patient study population and Kokoska 35. Silva and co-workers recommend lymphadenectomy for tumours with 10 or more mitoses per high-power field, in cases of lymphatic invasion, or when tumours are composed of small cells, that is, the small-cell subtype [132].

According to the literature on MCC, the sentinel lymphnode biopsy (SLNB) technique was used in approximately 100 cases between 1976 and 2002. Most reports advocate SLNB because morbidity is low, and because it provides an easy and reliable way of locating occult metastasis [102,133,134]. Recent studies have investigated the efficacy of SLNB in helping to determine whether lymph basin evacuation is necessary. These studies have been conducted on only a small series of patients, but even so, the results suggest that a negative sentinel node may obviate the need for neck dissection. SLNB is strongly advocated in the treatment and staging of individual tumours. Its use will improve the accrual of patients to adjuvant and further surgical treatment protocols [102,135]. Especially for the MCC cases occurring in the head and neck region the SLNB seems to be a safe and reliable technique for regional staging [136].

It is widely accepted that patients with regional node metastases or local or regional recurrence should undergo excision of the primary lesion and lymph node dissection [130]. Adjuvant radiation therapy to the primary site and regional nodes is generally recommended in addition to lymph node dissection. Tumours arising in the head and neck region with parotid gland metastasis seem to have even poorer prognosis [137]. As a result elective lymph node dissection has been recommended for younger patients with large tumours or tumours of the head and neck region [138-140].

Mohs micrographic surgery is a surgical technique developed by Frederick Mohs in the 1930s at the university of Wisconsin [141]. Briefly, the technique consists of debulking the tumour with a semisharp curette, in order to outline the skin tumour. Subsequently, a scalpel is used to remove the tissue in a horizontal fashion with 2 mm clinical margins. The tissue sample is taken to the laboratory for the processing. The patient is bandaged and waiting for the microscopic results. The horizontal frozen sections are cut from the undersurface of the tissue parallel to the skin surface, rather than vertically. This method allows for microscopic examination of the entire deep and peripheral margins of the surgical specimen. The surgeon does the microscopic examination. These steps are then repeated until the margins are free from the tumour, and the defect is closed.

Mohs micrographic surgery has been recommended as an advanced technique for local control, especially in areas calling for excellent cosmetic results (i.e. head and neck region) without compromising the principles of cancer surgery [142,143]. Then again, Brissett and co-workers reported inferior 2-year survival with patients undergoing Mohs micrographic surgery compared to patients who had wide local excision alone or wide local excision and lymphnode basin evacuation, 33%, 68% and 100% respectively [130].

#### Oncological treatment

Chemotherapy is generally reserved for stage III (distant metastasis) cases of MCC. The carcinoma has often been shown to respond to chemotherapy but, as in SCLC, remission is brief. Recently, Waldman and co-workers reported a complete remission that lasted for 6 months. The remission was achieved through the use of high-dose polychemotherapy following autologous blood stem cell transplantation [144]. Some months previously, Voog *et al *reported similar results, the rates of response to second-line (n = 33) and third-line (n = 10) chemotherapy being 45% and 20%, respectively [145]. No chemotherapeutic protocol has, however, been able to achieve a significant increase in survival rate [105].

No standard chemotherapy protocol has yet been established for the treatment of MCC. Because of the morphological and immunohistochemical similarity of MCC to SCLC, chemotherapy has been performed with protocols based largely on agents active in SCLC. A wide variety of chemotherapeutic agents, including the cytostatic drugs cyclophosphamide, doxorubicin, epirubicin, vincristine, etoposide, cisplatine, carboplastin, 5-fluorouracil, dacarbazine, mitoxantrone, bleomycine and iphosphamide, have been discussed [87,96,145-147]. Unfortunately, reports to date consist of only small studies and anecdotal evidence. A few reports have shown markedly high mortality among MCC patients receiving chemotherapy for metastatic disease. Tai and co-workers reported seven (3.4%) toxic deaths among 204 cases [96]. Voog and co-workers gave even higher mortality, the rate of toxic death during first-line treatment being 7.7% in 101 patients [145].

MCC is a highly radiosensitive tumour, which has been demonstrated in in vitro studies [148]. Many authors have recommended post-operative radiotherapy based on the retrospective comparison of patients treated with surgery alone with patients treated with surgery and post-operative radiotherapy [149,150]. In a recent series of 37 MCC patients Veness et al. reported a significantly longer median disease free survival with patients treated with surgery and adjuvant radiotherapy and than those undergoing surgery alone (23 months vs. 6 months) [151]. Moreover, outcome improved markedly in patients receiving adjuvant radiotherapy after surgery [152]. Radiation is most commonly used as an adjuvant therapy after surgery but it may be used to as the only treatment especially in patients who are too weak to undergo surgical treatments [153,154]

## Conclusion

Recent advances in the research of MCC have revealed features in the natural biology of this malignancy. However, several questions regarding the biology and pathology of the tumour remain to be addressed. The molecular events leading to oncogenic alterations must be identified. Mechanisms of metastasis are still largely concealed. Revealing these biological properties, hopefully, will lead to discovery of new treatments, e.g. the role of anti-angiogenic medication is still uncertain. Of the surgical treatments, the sentinel lymph node biopsy has proven its efficacy in finding the clinically occult metastasis and accrual of patients to adjuvant and further surgical and oncological treatment protocols.

Several markers for prognosis have been studied, but none have proven its value like large, ≥ 2 cm, primary tumour size. The wide range of the factors associated to survival may reflect the absence of large institutional studies.

## Competing interests

The author(s) declare that they have no competing interests.

## Authors' contributions

VK: concived the idea, did literature search, wrote the article and edited it for its final content.
